# Systemic inflammation early after kidney transplantation is associated with long-term graft loss: a cohort study

**DOI:** 10.3389/fimmu.2023.1253991

**Published:** 2023-10-02

**Authors:** Torbjørn F. Heldal, Anders Åsberg, Thor Ueland, Anna V. Reisæter, Søren E. Pischke, Tom E. Mollnes, Pål Aukrust, Finn Reinholt, Anders Hartmann, Kristian Heldal, Trond G. Jenssen

**Affiliations:** ^1^ Department of Internal Medicine, Telemark Hospital Trust, Skien, Norway; ^2^ Institute of Clinical Medicine, University of Oslo, Oslo, Norway; ^3^ Department of Transplantation Medicine, Oslo University Hospital – Rikshospitalet, Oslo, Norway; ^4^ Norwegian Renal Registry, Oslo University Hospital – Rikshospitalet, Oslo, Norway; ^5^ Department of Pharmacy, University of Oslo, Oslo, Norway; ^6^ K.G. Jebsen Thrombosis Research and Expertise Center, University of Tromsø, Tromsø, Norway; ^7^ Research Institute of Internal Medicine, Oslo University Hospital - Rikshospitalet, Oslo, Norway; ^8^ Department of Immunology, University of Oslo and Oslo University Hospital, Oslo, Norway; ^9^ Department of Anesthesiology, Division of Emergencies and Critical Care, Oslo University Hospital, Oslo, Norway; ^10^ Research Laboratory, Nordland Hospital Bodø, Bodø, Norway; ^11^ Section of Clinical Immunology and Infectious Diseases, Oslo University Hospital – Rikshospitalet, Oslo, Norway; ^12^ Department of Pathology, Oslo University Hospital, Rikshospitalet, Oslo, Norway; ^13^ Institute of Health and Society, University of Oslo, Oslo, Norway

**Keywords:** kidney transplantation, inflammation, graft failure, graft loss, biomarkers

## Abstract

**Background:**

Early graft loss following kidney transplantation is mainly a result of acute rejection or surgical complications, while long-term kidney allograft loss is more complex. We examined the association between systemic inflammation early after kidney transplantation and long-term graft loss, as well as correlations between systemic inflammation scores and inflammatory findings in biopsies 6 weeks and 1 year after kidney transplantation.

**Methods:**

We measured 21 inflammatory biomarkers 10 weeks after transplantation in 699 patients who were transplanted between 2009 and 2012 at Oslo University Hospital, Rikshospitalet, Norway. Low-grade inflammation was assessed with predefined inflammation scores based on specific biomarkers: one overall inflammation score and five pathway-specific scores. Surveillance or indication biopsies were performed in all patients 6 weeks after transplantation. The scores were tested in Cox regression models.

**Results:**

Median follow-up time was 9.1 years (interquartile range 7.6-10.7 years). During the study period, there were 84 (12.2%) death-censored graft losses. The overall inflammation score was associated with long-term kidney graft loss both when assessed as a continuous variable (hazard ratio 1.03, 95% CI 1.01-1.06, *P* = 0.005) and as a categorical variable (4^th^ quartile: hazard ratio 3.19, 95% CI 1.43-7.10, *P* = 0.005). In the pathway-specific analyses, fibrogenesis activity and vascular inflammation stood out. The vascular inflammation score was associated with inflammation in biopsies 6 weeks and 1 year after transplantation, while the fibrinogenesis score was associated with interstitial fibrosis and tubular atrophy.

**Conclusion:**

In conclusion, a systemic inflammatory environment early after kidney transplantation was associated with biopsy-confirmed kidney graft pathology and long-term kidney graft loss. The systemic vascular inflammation score correlated with inflammatory findings in biopsies 6 weeks and 1 year after transplantation.

## 1 Introduction

Early graft loss after kidney transplantation is mainly a result of acute rejection or surgical complications, while long-term graft loss is due to multifactorial conditions resulting in persistent pathological processes (1–5). Of the long-term events, more than 50% of graft losses are caused by more than one contributing factor (5). Improvement in short-term graft survival has been superior to the improvement of long-term graft survival in the last decades, and the high degree of long-term graft failure is not satisfactory (6, 7). The most important causes of long-term graft loss are preceding acute rejection episodes including both T-cell-mediated and antibody-mediated rejections, calcineurin inhibitor (CNI) toxicity, infections, and recurrence of the primary kidney disease (8, 9). Additionally, non-specific chronic injury and nephron loss potentially caused by diverse drug toxicity, metabolic derangements, graft cancer, and other yet unknown causes are relevant for long-term graft loss.

Kidney transplant recipients are characterized by an activated immune response towards the allograft, contributing to low-grade persistent inflammation in these patients that may be reflected at the systemic level (10, 11). Low-grade inflammation is an established risk factor for mortality in both the general healthy population (12) and among kidney transplant recipients (13–15). Inflammatory biomarkers, such as high-sensitive C-reactive protein (CRP), interleukin-6, and terminal C5b-9 complement complex (TCC), have been described to be associated with long-term graft loss (15, 16). Inflammation in early biopsies after kidney transplantation is associated with the progression of interstitial fibrosis, reduced kidney graft function, and development of *de novo* donor-specific antibodies (dnDSA) (17–19). Key histological features in a rejection are microvascular-, tubular-, and interstitial inflammation with the presence of a variety of immune cells reflecting activation of several pathways (20–23), but if this is reflected at the systemic level is still unclear.

An obvious goal after transplantation is to maximize the longevity of the graft, and thus it is important to develop tools for the identification of patients at high risk for graft loss and explore potential novel therapeutic targets for reducing such risks. In the present study, using previously defined inflammation scores developed in the same population (14), we tested the hypothesis that there is an association between the degree and pattern of systemic inflammation in the early period after kidney transplantation and death-censored long-term kidney graft loss. We also examined the associations between systemic inflammation scores and local findings in biopsies 6 weeks and 1 year after transplantation.

## 2 Materials and methods

### 2.1 Study population and design

**Figure 1:**
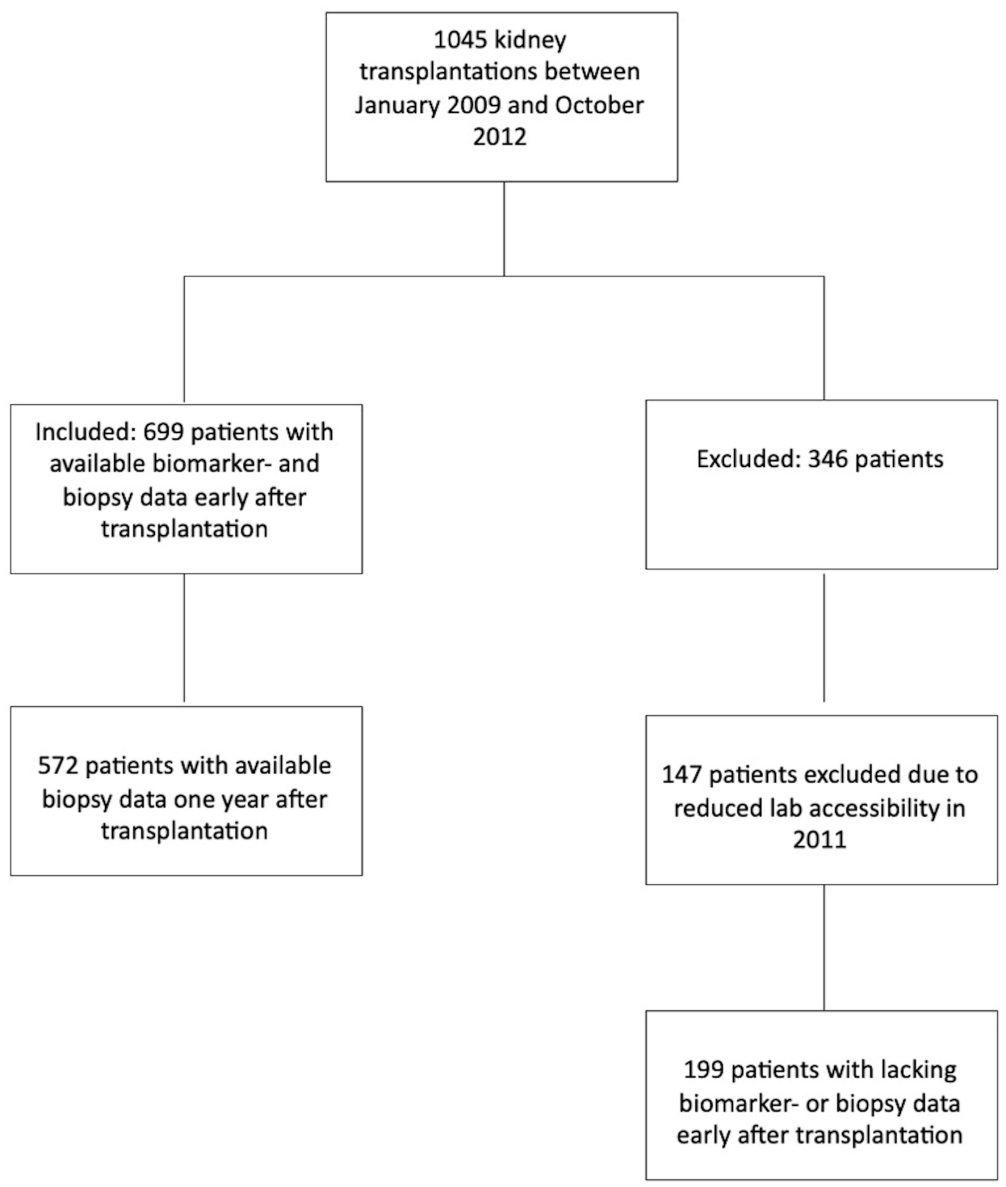
Flow chart; overview of included and excluded patients.

In this cohort study, we included 699 adult patients who underwent kidney transplantation at Oslo University Hospital, Rikshospitalet, Norway, between January 2009 and October 2012 (**Figure 1**). No patients were lost to follow-up. Patients without ongoing verified infections underwent a surveillance assessment 10 weeks after transplantation, where 21 inflammatory and related biomarkers were measured in samples stored under optimal conditions. The study was approved by the Regional Ethics Committee in Norway and was performed in accordance with the Helsinki Declaration.

At the 10-week posttransplantation follow-up, all patients, except for those with preexisting diabetes mellitus or fasting glucose levels > 7.0 mmol/L, performed an oral glucose tolerance test (OGTT). Posttransplant diabetes mellitus (PTDM) was diagnosed according to the modified ADA criteria (24): fasting plasma glucose concentration ≥ 7.0 mmol/L, or two-hour plasma glucose concentration ≥ 11.1 mmol/L.

### 2.2 Immunosuppressive protocol

The immunosuppressive protocol has been described in detail previously (14). In short, the standard immunosuppressive therapy during the study period consisted of induction treatment with methylprednisolone and the IL-2 receptor antibody basiliximab, followed by maintenance treatment combining glucocorticoids, the cell proliferation inhibitor mycophenolate, and a CNI. High immunological risk patients were defined as patients with preformed DSA at the time of transplantation or patients receiving an ABO-incompatible transplant. These patients received induction treatment consisting of intravenous human immunoglobulin and rituximab in addition to methylprednisolone and basiliximab. Kidney transplant recipients with immunological intermediate risk (complement-dependent cytotoxicity (CDC) panel reactive antibody (PRA) positive (>20%)) received methylprednisolone and anti-thymocyte globulin (ATG) as induction treatment.

Target trough levels for CNI concentrations in immunological standard risk patients were for tacrolimus 3-7 μg/L from the day of engraftment. For cyclosporine, the initial target was 200-300 μg/L, and then 75-125 μg/L after 6 months. In intermediate and high-risk patients, the initially targeted trough levels were 10-12 μg/L during the first month for tacrolimus and 250-350 μg/L for cyclosporine, and throughout the rest of the first year 6-10 μg/L for tacrolimus and 150-250 μg/L for cyclosporine. After the first year, the targeted levels were 5-8 μg/L for tacrolimus and 100-175 μg/L for cyclosporine. Prednisolone dosage was tapered to 5 mg/day 6 months posttransplant. Patients treated with tacrolimus received 750 mg mycophenolate mofetil or 540 mg mycophenolate sodium twice daily, while patients who received cyclosporine were treated with 1000 mg mycophenolate mofetil or 720 mg mycophenolate sodium twice daily. The standard rejection treatment was SoluMedrol 1375 mg divided into five dosages and in case of steroid-resistant rejection ATG was given. Banff borderline rejection in indication biopsies received rejection treatment, while borderline findings in protocol biopsies did not.

### 2.3 Measurement of inflammatory biomarkers, anti-human leukocyte antigen antibodies, and HLA mismatches

The 21 inflammatory biomarkers have previously been described in detail (14, 25). They are listed sequentially in **Table 1**. Blood samples obtained during the in-depth investigation 10 weeks after kidney transplantation were stored in a biobank at -80^0^C for research purposes. All analyses in this study were performed in one batch on these samples. The inflammatory biomarkers retrieved at the 10-week follow-up visit were measured in plasma or serum by enzyme immunoassays (EIA) using commercially available antibodies (R&D Systems, Minneapolis, MN) in a 384-well format using a combination of a SELMA (Jena, Germany) pipetting robot and a BioTek (Winooski, VT) dispenser/washer. Absorption was read at 450 nm with wavelength correction set to 540 nm using an EIA plate reader (Bio-Rad, Hercules, CA). TCC was measured by EIA using a monoclonal antibody aE11 reacting with a neoepitope exposed in C9 when incorporated in TCC (26). Intra- and inter-assay coefficients of variation were < 10% for all assays (27), and the coefficients of variation were based on the performance in the laboratory that set up these analyses. The samples were thawed less than three times. In the final study population, no patients had values below the level of detection.

**Table 1:** Overview of inflammation scores and associated inflammatory biomarkers.

Overall inflammation score	Fibrogenesis activity	General/vascular inflammation	Metabolic inflammation	Growth and angiogenesis activity	Leukocyte activity
GDF-15^1^ CXCL16 sTNFR1 MIF PTX3 YKL40 Granulysin IGFBP1 Periostin NGAL TCC	GDF-15 Syndecan Osteopontin Cathepsin S Periostin	CXCL16 sTNFR1 PTX3 TCC	IGFBP1 Resistin IGF-1 Chemerin	GAS6 AXL6 EPCR	MIF Granulysin NGAL YKL40

^1^GDF-15, Growth differentiation factor 15; CXCL16, CXC chemokine ligand 16; sTNFR1, soluble tumor necrosis factor receptor 1; MIF, Macrophage inhibitory factor; PTX3, Pentraxin 3; YKL40, Tyrosine; lysine; leucine – 40; IGFPB1, insulin-like growth factor binding protein 1; NGAL, Neutrophil gelatinase-associated lipocalin; TCC, Terminal 5b-9 complement complex; IGF-1, insulin-like growth factor-1; GAS6, growth arrest-specific gene 6; AXL6, receptor tyrosine kinase 6; EPCR, endothelial protein C receptor.

Complement-dependent cytotoxicity crossmatches were performed with recipient serum and donor peripheral blood T cells and B cells in all patients at transplantation. Testing for HLA antibodies was done as complement-dependent cytotoxicity PRA and with One Lambda Labscreen^®^ mixed and single antigen beads with Luminex^®^ and HLA Fusion software (One Lambda Inc., Canoga Park, CA). A normalized mean fluorescent intensity cutoff point of 1000 was used for positivity in single antigen analyses. Testing was done pretransplant, at transplantation, 6 weeks and one 1 year after transplantation, and at the time of indication biopsy (17). HLA typing in recipients and living kidney donors was primarily performed with sequence-specific oligonucleotide (SSO, OnleLambda, CA, USA) and re-typing with Sanger sequencing (SPT, Utrecht, The Netherlands). Deceased donors were typed primarily with RT-PCR (SSP, Olerup CareDx, CA, USA) technique and repeated with SSO. HLA mismatches are reported on broad serological antigens (first field).

### 2.4 Biopsies and histological classification

The biopsy procedure for this population has been described earlier (17). Protocol biopsies were performed 6 weeks and 1 year after transplantation. Cores for histology and for C4d were obtained with ultrasound guidance using an 18-gauge biopsy gun. For reliable biopsy analyses the goal was to obtain a minimum of seven glomeruli and one artery in total per patient, and with the optimal goal of ten glomeruli and two arteries or more. Tubulointerstitial tissue was graded according to interstitial inflammation (i), tubulitis (t), interstitial fibrosis (ci), and tubular atrophy (ct). Inflammation in areas with interstitial fibrosis, tubular atrophy, and total inflammation were not graded by the pathologists. C4d was stained with indirect immunofluorescence on frozen sections (monoclonal antibody, Quidel, San Diego, CA).

The kidney lesions were originally graded according to Banff 2009 guidelines by the pathologists (17, 28), whereas criteria from the Banff 2019 were implemented to diagnose rejection. The biopsies were allocated into one of four groups: 1) no inflammation and no interstitial fibrosis and tubular atrophy (IFTA) (i+t ≤ 1 and ci+ct ≤ 1), (2) isolated inflammation (i+t ≥ 2 and ci+ ct ≤ 1), (3) IFTA and no inflammation (i+t ≤ 1 and ci+ct ≥ 2), and (4) inflammation + IFTA (i+IFTA) (i+t ≥ 2 and ci+ct ≥ 2).

### 2.5 Inflammation scores

Six composite inflammation scores were constructed prior to the analyses based on the principles used for the INFLA score in the Moli-sani study (12). The development of the inflammation scores has been previously described in detail (14), and an overview of these scores is given in **Table 1**. Deciles were generated for each biomarker. Values within the four highest deciles scored 1 to 4, while the four lowest deciles scored -4 to -1. The 5^th^ and 6^th^ scored 0 points. The scores for each inflammatory biomarker were summed up. The total score was then divided into quartiles for graft survival association analyses. In addition to an overall inflammation score including 11 biomarkers, five pathway-specific inflammatory scores representing increased (i) fibrogenesis, (ii) vascular/general inflammation-, (iii) metabolic inflammation, (iv) cellular growth/angiogenesis, and (v) leukocyte activation were constructed. The different scores were based on the values of 3-5 biomarkers. In addition, one *post hoc* inflammation score was created based on the individual inflammatory biomarkers with the highest effect estimates related to reduced death-censored graft survival (osteopontin, GDF-15, sTNFR1, and NGAL). Insulin-like growth factor binding protein 3 (IGFBP3) was only tested as an individual biomarker and was not included in any of the composite scores to avoid covariance due to its close relationship with IGF-1 (see subsection statistical analyses). The distribution of the standardized individual biomarkers is presented in **Supplementary Figure 1**.

### 2.6 Outcomes

The primary outcome was death-censored kidney graft loss. This was defined as either a return to dialysis or kidney retransplantation. Patients who died with a functioning allograft were censored at the time of death as functioning allograft. Secondly, we also investigated associations between systemic inflammation and findings in kidney graft biopsies, and in these analyses, we used inflammation and IFTA as the outcome variables. Survival and graft loss data were retrieved from the Norwegian Renal Registry on 23 December 2020.

### 2.7 Statistical analyses

The statistical analyses were performed in StataCorp Stata/SE 17.0. We used two-sided hypothesis tests, and the significance level was set at 0.05. Continuous variables were described by using means and standard deviations, and categorical variables by proportions. We compared means and proportions between the different inflammatory groups by one-way ANOVA and chi-square tests. Kaplan-Meier plots were created for the different inflammatory groups to estimate graft survival, and differences were tested by using log-rank tests. The effect of inflammation scores on kidney graft loss was explored using Cox regression models, with long-term kidney graft loss as the dependent variable. For death-censored kidney graft loss, recipients were censored at the time of death with a functioning graft or on 23 December 2020. The proportional-hazards assumptions were tested by PH-tests. The models were adjusted for recipient and donor age, body mass index (BMI), number of transplantations, PRA-positivity, number of HLA-DR mismatches, cold ischemia time, dialysis vintage, estimated glomerular filtration rate (eGFR_CKD-EPI_) at baseline, deceased (vs. living) donor, immunological high risk, DGF, DSA, rejection including subclinical rejection in biopsies 6 weeks after transplantation, smoking status, and pretransplant or posttransplant diabetes mellitus (within the first 10 weeks). Considering HLA mismatches, the initial models were tested with both the total number of HLA A mismatches, B mismatches, and DR mismatches, and isolated increased HLA DR mismatches, which were included in the final analysis as there were no differences between the two models. PRA levels above 20% were considered positive. The value of each biomarker was standardized by dividing the real value by the standard deviation, and the standardized value was then tested in the Cox regression model. In the analyses with acknowledged DSA at any time during the first year, a new baseline was set to one year after transplantation, and patients who experienced graft loss within the first year were excluded. Finally, we also performed multivariable logistic regression models with IFTA and i+IFTA in biopsies one year after transplantation as outcome variables, adjusted for age, donor age, ischemic time, number of HLA DR mismatches, PRA, type of CNI, DGF, preformed or dnDSA within the first year, and inflammation scores. Correlations between the inflammation scores and i-, t-, ct-, ci-, g-, and ptc-scores in biopsies were examined by linear regression models including the same variables as in the multivariable logistic regression model described above. For determination of the association between IFTA and i+IFTA in biopsies 6 weeks after transplantation and systemic inflammation 10 weeks after transplantation, we performed a linear regression model with the continuous vascular inflammation score and the fibrogenesis score as the outcome variables (adjusted for the risk factors described above).

In all models, the inflammation scores were included both as continuous and categorical variables, and each standardized biomarker was tested in the model. When the inflammation scores were tested as a categorical variable, the 1^st^ quartile of the inflammation score was used as the reference category.

## 3 Results

### 3.1 Study population and biopsy findings

Baseline characteristics are presented in **Table 2**. The median follow-up time for death-censored kidney graft loss was 9.1 years (interquartile range 7.6-10.7 years). During the study period, there were 84 (12.2%) death-censored graft losses. Of these events, 57 (67.9%) were due to biopsy-verified rejection while on immunosuppressive medication, 13 (15.5%) occurred after the recurrence of the primary kidney disease, 4 (4.8%) after *de novo* glomerulonephritis, and 10 (11.9%) were due to other causes. At the time of transplantation, 59 (8.4%) patients had performed DSA, 26 (3.7%) developed dnDSA during the first 6 weeks, and 44 (6.3%) had dnDSA 1 year following transplantation. In total, 129 (18.5%) had either preformed DSA or dnDSA during the first year.

**Table 2:** Clinical baseline characteristics according to quartiles of the overall inflammation score^1^.

	1^st^ Quartile (N=162 (23.2%))	2^nd^ Quartile (N=172 (24.6%))	3^rd^ Quartile (N=177 (25.3%))	4^th^ Quartile (N=189 (27.0%))	P-value
Age (years)	44 (14)	52 (14)	55 (13)	60 (11)	< 0.001
Sex (male)	96 (59.3%)	121 (70.3%)	132 (74.6%)	134 (70.9%)	0.021
Donor age (years)	44 (14)	48 (15)	51 (17)	56 (15)	< 0.001
BMI^2^ (kg/m^2^)	24 (3.6)	26 (13.6)	26 (4.1)	26 (4.6)	0.164
eGFR (ml/min/1.73m^2^)	75 (18)	64 (18)	58 (19)	47 (18)	< 0.001
Cold ischemia (hours)	8.6 (6.8)	9.2 (6.5)	10.4 (5.9)	11.5 (5.9)	< 0.001
Dialysis vintage (months)	10 (14)	11 (14)	15 (16)	18 (16)	< 0.001
Deceased donor	84 (51.9%)	103 (59.9%)	140 (79.1%)	157 (83.1%)	< 0.001
Multiple transplants (>1)	16 (9.9%)	24 (14.0%)	27 (15.3%)	31 (16.4%)	0.331
HLA DR mismatches (≥1)	104 (64.2%)	106 (61.6%)	114 (64.4%)	120 (63.5%)	0.963
CDC-PRA (> 20%)	5 (3.1%)	6 (3.5%)	6 (3.4%)	8 (4.2%)	0.770
DGF	6 (2.5%)	6 (2.4%)	15 (5.8%)	32 (12.6%)	< 0.001
Tacrolimus	126 (77.8%)	102 (59.3%)	94 (53.1%)	91 (48.1%)	< 0.001
Cyclosporine	34 (21.0%)	69 (40.1%)	78 (44.1%)	97 (51.3%)	< 0.001
Preformed DSA	11 (6.8%)	20 (11.6%)	11 (6.2%)	17 (9.0%)	0.218
dnDSA 6 weeks after transplantation	5 (3.1%)	9 (5.2%)	7 (4.0%)	5 (2.6%)	0.298
dnDSA 1 year after transplantation	12 (7.4%)	12 (7.0%)	14 (7.9%)	6 (3.2%)	0.347
Pretransplant DM	22 (13.6%)	27 (15.7%)	52 (29.4%)	61 (32.3%)	< 0.001
PTDM	13 (8.0%)	5 (2.9%)	12 (6.8%)	17 (9.0%)	< 0.001
Current smoker	32 (19.8%)	36 (20.9%)	45 (25.4%)	35 (18.5%)	0.758

^1^Continuous variables are presented as means with the following standard deviations, and categorical variables are presented as absolute numbers and percentages.

^2^BMI, body mass index; eGFR, estimated glomerular filtration rate; CDC, complement-dependent cytotoxicity; PRA, panel-reactive antibodies; DGF, delayed graft function; DSA, donor-specific HLA-antibodies; dnDSA, denovo donor-specific HLA-antibodies; DM, diabetes mellitus; PTDM, post-transplant diabetes mellitus.

Early biopsies 6 weeks after transplantation were performed in 699 patients (601 (86.0%) protocol biopsies and 98 (14.0%) indication biopsies), and biopsies 1 year after transplantation were performed in 574 patients. Of these, 557 (97.0%) were protocol biopsies and 17 (3.0%) were indication biopsies. Biopsy findings 6 weeks after transplantation are presented in **Table 3**. Within the first 6 weeks after transplantation, 76 (10.9%) patients experienced biopsy-proven acute rejection (BPAR) or received treatment on clinical indication with negative biopsy findings. Of these, 50 (65.8%) were classified as Banff 1A or higher, and 18 (23.7%) had Banff borderline changes with a rise in creatinine. In all, 8 (10.5%) patients were treated for rejection without significant biopsy findings. BK-virus nephropathy was present in only one patient, while there were no cases of cytomegalovirus nephropathy.

**Table 3:** Biopsy findings 6 weeks after kidney transplantation in patients treated for acute rejection per quartile of the overall inflammation score.

	1^st^ quartile (n=162 (23.2%))	2^nd^ quartile (n=172 (24.6%))	3^rd^ quartile (n=177 (25.3%))	4^th^ quartile (n=189 (27.0%))	P-value
Treated for rejection^1^	15 (9.3%)	13 (7.6%)	25 (14.1%)	23 (12.2%)	0.466
BPAR (indication)	4 (2.5%)	5 (2.9%)	2 (1.1%)	6 (3.2%)	–
BPAR (protocol)	9 (5.6%)	5 (2.9%)	13 (7.3%)	6 (3.2%)	–
Borderline + graft dysfunction	2 (1.2%)	2 (1.2%)	6 (3.4%)	8 (4.2%)	–
Borderline	35 (21.6%)	35 (20.3%)	41 (23.2%)	48 (25.4%)	0.574
Acute AMR	0 (0.0%)	1 (0.6%)	0 (0.0%)	1 (0.5%)	0.572
Chronic AMR	1 (0.6%)	0 (0.0%)	1 (0.6%)	0 (0.0%)	0.813
C4d positivity	6 (3.7%)	5 (2.9%)	4 (2.3%)	8 (4.2%)	0.154
TCMR					
1A	7 (4.3%)	4 (2.3%)	7 (4.0%)	9 (4.8%)	0.674
1B	4 (2.5%)	3 (1.7%)	5 (2.8%)	2 (1.1%)	0.681
2A	2 (1.2%)	2 (1.2%)	2 (1.1%)	2 (1.1%)	–
2B	1 (0.6%)	1 (0.6%)	1 (0.6%)	1 (0.5%)	–
3	–	–	–	–	–

^1^Total number of patients treated for rejection within the first 6 weeks.

BPAR, biopsy-proven acute rejection; TCMR, T-cell mediated rejection; AMR, antibody-mediated rejection.

### 3.2 Death-censored kidney graft loss: multivariable Cox regression analyses

#### 3.2.1 Overall inflammation score

**Figure 2:**
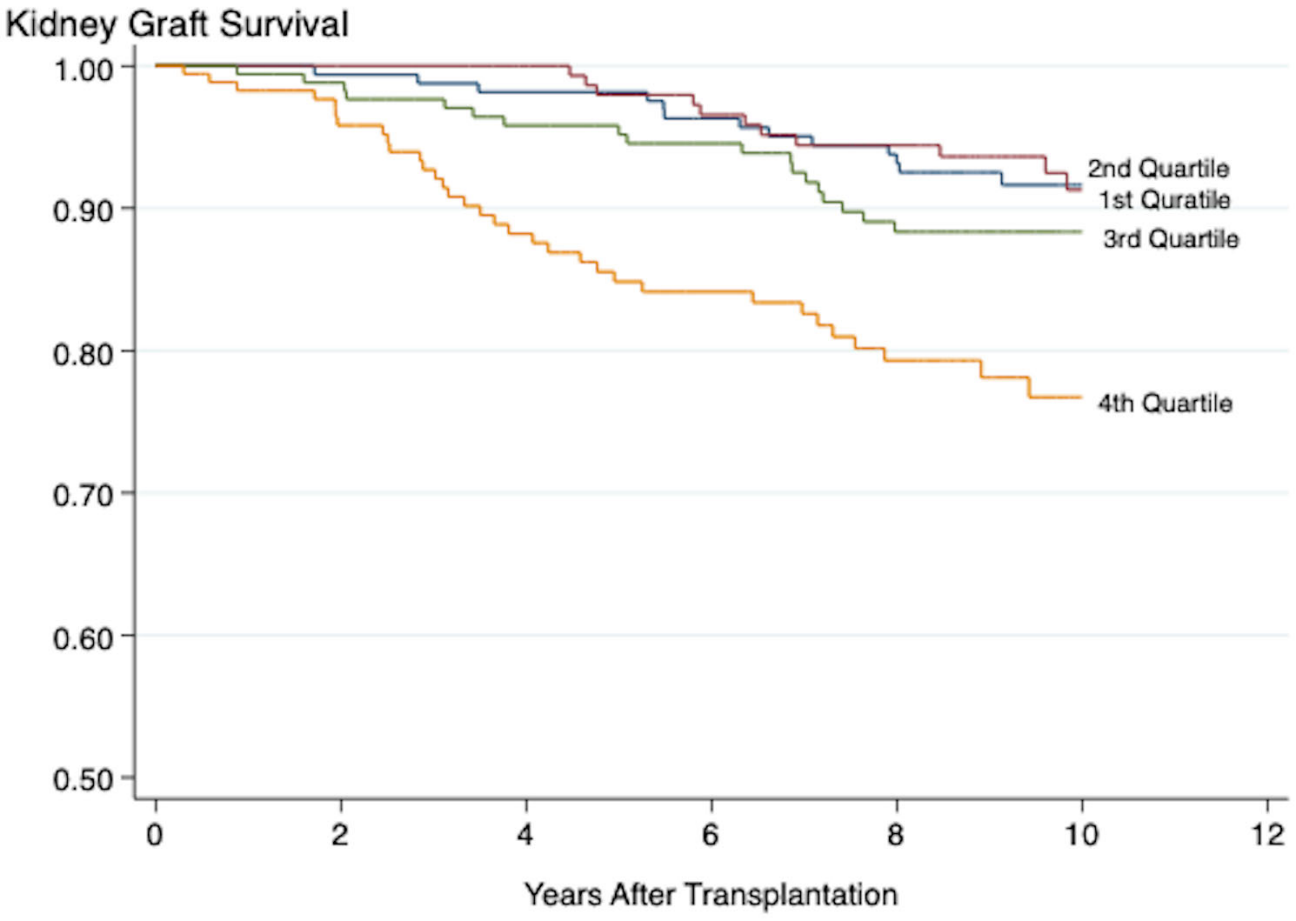
Kaplan-Meier plot showing the association between the overall inflammation score and death-censored kidney graft survival. Log-Rank < 0.001. Plots marked 1^st^-4^th^ quartile represent patients with increasing grade of inflammation.

When tested as a continuous variable, the overall inflammation score was associated with long-term death-censored kidney graft loss (hazard ratio (HR) 1.03, 95% CI 1.01-1.06, *P* 0.005). The results were consistent also when the score was assessed as a categorical variable using the 1^st^ quartile as reference (2^nd^ quartile HR 1.03, *P* 0.952, 3^rd^ quartile 1.61, *P* 0.250, and 4^th^ quartile 3.19 P 0.005) (**Figure 2**, **Table 4**). Recipient age (HR 0.97, 95% CI 0.95-0.99, *P* 0.005), eGFR at baseline (HR 0.97, 95% CI 0.95-0.98, *P* < 0.001), number of HLA-DR mismatches (HR 2.21, 95% CI 1.27-3.84, *P* 0.005), CDC-PRA >20% (HR 9.08, 95% CI 1.67-49.29, *P* 0.011), current smoking at transplantation (HR 2.06, 95% CI 1.22-3.49, *P* 0.007), and preformed DSA (HR 2.36, 95% CI 1.23-4.53, P 0.010) were all significantly associated with death-censored kidney graft loss.

**Table 4:** Cox regression analysis of risk factors including pathway-specific inflammation scores and death-censored kidney graft loss adjusted for risk factors^1,2^.

Pathway-specific inflammation score	Score as a continuous variable (HR, 95% CI, p-value)	2^nd^ Quartile (HR, 95% CI, p-value)	3^rd^ Quartile (HR, 95% CI, p-value)	4^th^ Quartile (HR, 95% CI, p-value)
Overall inflammation	1.03, (1.01-1.06, p=0.005)	1.03 (0.46-2.29, p=0.952)	1.61 (0.72-3.60, p=0.250)	3.19 (1.43-7.10, p=0.005)
Fibrogenesis	1.07 (1.02-1.11, p=0.003)	2.05 (0.91-4.65, p=0.085)	1.92 (0.81-4.54, p=0.136)	2.90 (1.12-7.03, p=0.018)
Vascular/general inflammation	1.07 (1.03-1.11, p<0.001)	1.73 (0.81-3.73 p=0.160)	3.14 (1.45-6.79, p=0.004)	3.31 (1.59-6.91, p=0.001)
Metabolic inflammation	1.06 (1.01-1.12, p=0.016)	0.79 (0.34-1.84, p=0.585)	0.93 (0.43-2.02, p=0.847)	1.79 (0.84-3.82, p=0.133)
Growth-/angiogenesis	1.02 (0.97-1.08, p=0.467)	1.06 (0.55-2.06, p=0.855)	1.05 (0.54-2.06, p=0.883)	1.10 (0.58-2.11, p=0.764)
Leukocyte activation	1.02 (0.97-1.06, p=0.441)	1.38 (0.69-2.75, p=0.361)	1.58 (0.78-3.20, p=0.204)	1.45 (0.70-3.03, p=0.320)

^1^The model adjusted for age, donor age, BMI, type of CNI, living or deceased donor, dialysis vintage, cold ischemic time, egfr at baseline, HLA-DR mismatches, CDC-PRA, number of transplantations, smoking status, pre- or post-transplant diabetes mellitus, DGF, DSA at transplantation, and early rejection.

^2^Hazard ratios are relative to the 1^st^ quartile of the inflammation scores.

In the model adjusted for dnDSA 6 weeks after transplantation, dnDSA had an HR of 3.09 (95% CI 1.01-1.06, *P* 0.011). The continuous inflammation score had an HR 1.03 (95% CI 1.01-1.06, *P* 0.002), and while assessed as a categorical variable the 4^th^ quartile of the inflammation score had an HR 3.30 (95% CI 1.51-7.17, *P* 0.003). When the model was adjusted for patients with either preformed DSA or dnDSA at any time during the first year after transplantation, the continuous inflammation score still had an HR of 1.04 (95% CI 1.01-1.06, *P* 0.001), and the 4^th^ quartile of the inflammation score had an HR of 3.27 (95% CI 1.49-7.16, *P* 0.003).

When the multivariable model also adjusted for inflammatory findings in the 6-week biopsies in addition to the systemic inflammation score, the systemic overall inflammation score remained significantly associated with death-censored graft loss (4^th^ quartile: HR 3.08, 95% CI 1.41-6.76, *P* 0.006), while local inflammation in the graft was not associated with graft loss. In a model consisting of patients who performed a 1-year biopsy, thus excluding patients with graft loss prior to this, the 4^th^ quartile of the overall inflammation score was significantly associated with graft loss (HR 6.03, 95% CI 2.27-16.03, *P* < 0.001). When graft inflammation was also included in the model, the overall systemic inflammation score (HR of 5.36, 95% CI 2.02-14.20, *P* < 0.001) and graft inflammation (HR 3.22, 95% CI 1.81-5.71 P *<* 0.001) were both significantly associated with graft loss.

#### 3.2.2 Pathway-specific inflammation scores

**Figure 3:**
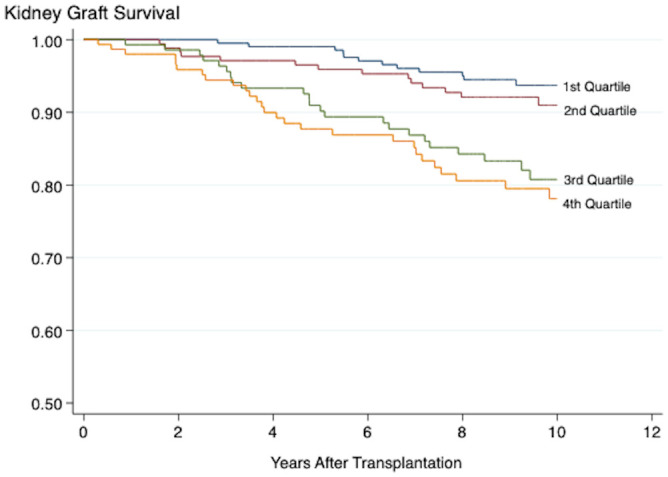
Kaplan-Meier plot showing the association between the vascular inflammation score and death-censored kidney graft survival. Log-Rank < 0.001. Plots marked 1^st^-4^th^ quartile represent patients with increasing grade of inflammation.

**Figure 4:**
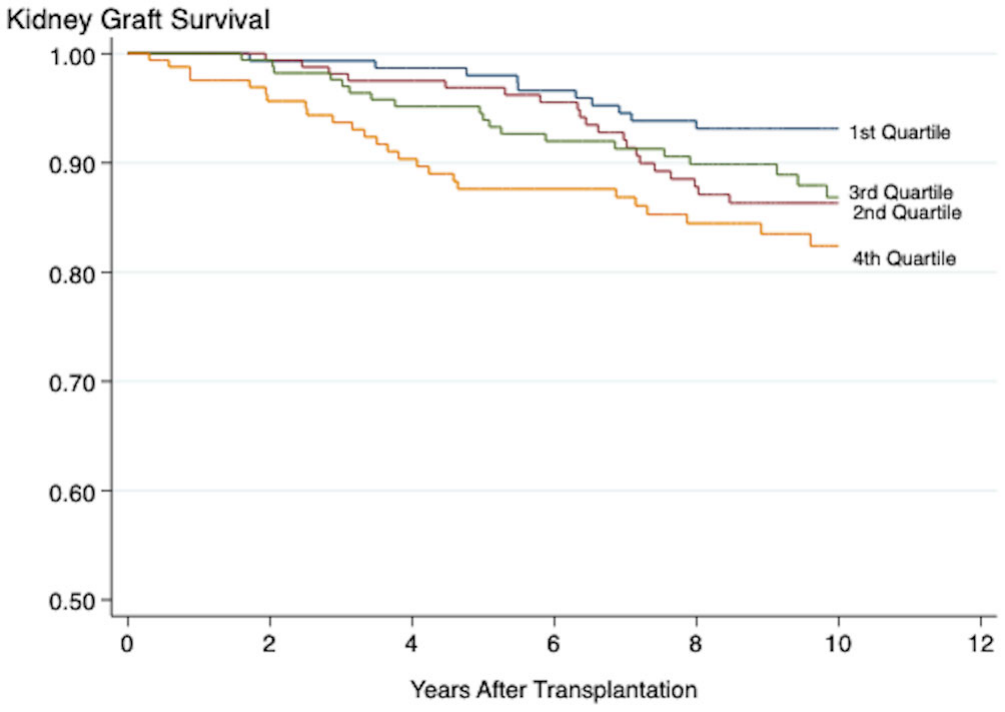
Kaplan-Meier plot showing the association between the fibrogenesis score and death-censored kidney graft survival. Log-Rank < 0.001. Plots marked 1^st^-4^th^ quartile represent patients with increasing grade of inflammation.

The results from the pathway-specific analyses are presented in **Table 4**. Of the five inflammation scores, only the vascular/general inflammation (4^th^ quartile: HR 2.83, 95% CI 1.59-6.91, *P <*0.001) and the fibrogenesis activity (4^th^ quartile: HR 2.05, 95% CI 1.12-7.03, *P* 0.020) scores were significantly associated with death-censored graft loss both when the scores were assessed as continuous and categorical variables (**Figures 3**, **4**). The continuous metabolic score was associated with graft loss, however, not as a categorical variable.

#### 3.2.3 Individual inflammatory biomarkers

The values of all 21 biomarkers were standardized and then tested individually in both univariable and multivariable Cox regression models. Resistin (HR 1.32, *P* < 0.001), osteopontin (HR 1.41, *P* < 0.001), GDF-15 (HR 1.42, *P* < 0.001), sTNFR1 (HR 1.51, *P* < 0.001), CXCL16 (HR 1.30*, P* 0.001), YKL40 (HR 1.80, *P* 0.041), chemerin (HR 1.29, *P* 0.001), and NGAL (HR 1.50, *P* < 0.001) all showed significant positive associations to death-censored graft loss. In the multivariable analyses adjusted for traditional risk factors and the significantly associated inflammatory biomarkers, osteopontin (fibrogenesis) (HR 1.30, *P* 0.050), GDF-15 (fibrogenesis) (HR 1.62, *P* < 0.001), and sTNFR1 (general/vascular inflammation) (HR 1.27, *P* 0.014) were significantly associated with death-censored graft loss.

### 3.3 Associations between inflammation scores and biopsy findings

An overview of the histological findings 6 weeks after transplantation is presented in **Table 3**. The histological diagnosis at 6 weeks was: no inflammation or IFTA (n=267), isolated inflammation (n=40), IFTA (n=285), and i+IFTA (n=105). At 1 year after transplantation, the histological diagnosis was: no inflammation or IFTA (n=194), inflammation (n=6), IFTA (n=263), and i+IFTA (n=111). In the multivariable linear regression model with the continuous vascular inflammation score as the outcome variable, i+IFTA at 6 weeks (β 2.21, *P* 0.001), DGF (β 3.50, *P* < 0.001), cyclosporine usage (β 1.72, *P* 0.002), age (β 0.08, *P <* 0.001), and donor age (β 0.05, P 0.001) showed significant positive associations.

In the multivariable logistic regression model using i+IFTA at 1 year as the dependent variable, donor age, DSA within the first year after transplantation, graft inflammation at 6 weeks, and the vascular inflammation score were statistically significantly associated with graft inflammation (**Table 5**). Inflammation scores representing other pathways did not come out statistically significant regarding i+IFTA.

**Table 5:** Multivariable logistic regression model showing associations between predictors and inflammation + IFTA in biopsies 1 year after kidney transplantation.

	OR	95% CI	P-value
Age (years)	0.99	0.97-1.02	0.648
Donor age (years)	1.02	1.00-1.04	0.025
Ischemic time (hours)	1.02	0.98-1.05	0.411
HLA DR mismatches (n)	1.58	0.96-2.59	0.070
CDC-PRA (> 20%)	1.15	0.37-3.60	0.813
Cyclosporine (yes)	1.14	0.68-1.90	0.630
Delayed graft function (yes)	1.21	0.65-39.62	0.646
dnDSA at the time of biopsy	1.86	1.04-3.34	0.038
i-IFTA at 6 weeks	2.78	1.61-4.82	<0.001
Vascular inflammation score (continuous)	1.04	1.01-1.08	0.035
Vascular inflammation score (categorical – 4^th^ quartile)	2.21	1.15-4.25	0.018

HLA, human leukocyte antigens; PRA, panel reactive antibodies; dnDSA, de novo donor-specific antibodies.

The i-score (β 1.32, *P* < 0.001), t-score (β 1.32, *P* < 0.001), and g-score (β3.56, *P* 0.001) in biopsies 6 weeks after transplantation correlated with the systemic vascular inflammation score in multivariable linear regression models. The same associations were observed 1 year after transplantation for the vascular inflammation score when the histological findings were used as the outcome variable (i-score: β 0.016, *P* 0.001, t-score: β 0.016, *P* 0.002). The vascular score was associated with the ptc-score in univariate analyses, but not in the multivariable models. IFTA in biopsies 6 weeks after transplantation correlated with the fibrogenesis score (β 1.27 P 0.010), and the fibrogenesis score was associated with IFTA in biopsies after 1 year (OR 1.04, *P* 0.011). The continuous fibrogenesis score correlated with the ci-score (β 0.012, *P* 0.014) and ct-score (β 0.011, *P* 0.014) 1 year after transplantation, but this was only the case for the ci-score (β 0.010, *P* 0.006) 6 weeks after transplantation.

## 4 Discussion

Our findings describe strong associations between subclinical low-grade systemic inflammation in the early phase after transplantation and death-censored graft loss, especially for markers reflecting fibrogenesis activity and vascular inflammation. This is in line with our previous findings showing that long-term mortality rates following kidney transplantation were also associated with increased levels of several inflammatory biomarkers (14). Kidney transplant recipients with the highest grades of systemic subclinical inflammation had three times increased risk of experiencing isolated kidney graft loss during the follow-up period. The overall inflammation score showed the highest effect estimates, but notably, increased fibrogenesis and vascular inflammation stood out among the pathway-specific scores and were also associated with biopsy findings both 6 weeks and 1 year after transplantation.

The score representing vascular inflammation was associated with inflammatory changes in biopsies both 6 weeks and 1 year after transplantation, and it correlated with interstitial inflammation, tubulitis, and glomerulitis. Of the individual biomarkers representing vascular inflammation, only sTNFR1, as a marker of activity in the TNF system, was associated with inflammation both 6 weeks and 1 year after transplantation, whereas the chemokine CXCL16 and the pentraxin PTX3 were only associated with inflammation in biopsies 6 weeks after transplantation. sTNFR1 was also associated with long-term death-censored kidney graft loss. Microvascular and macrovascular inflammation are central findings in biopsies from the kidney grafts during rejection (20, 21), but microvascular abnormalities may be present without acute rejection episodes and these types of subclinical changes are observed in chronic rejection (20, 21, 29). Our results are in line with these observations, as the score representing vascular inflammation, and in particular, sTNFR1 stood out among the pathway-specific analyses of death-censored graft loss.

Fibrogenesis and inflammation are two interacting processes related to chronic kidney disease, and biopsy studies have shown activation of such processes in kidney graft rejection (30). In the present study, we show that systemic markers of fibrogenesis, in particular GDF-15 and osteopontin, in addition to the fibrogenesis score, were independently associated with death-censored kidney allograft loss. The fibrogenesis score was also associated with IFTA in biopsies 6 weeks and 1 year after transplantation, and the score correlated with both interstitial fibrosis and tubular atrophy. The interpretation of findings in biopsies early after transplantation is complicated as it is difficult to determine if the findings are donor- or surgery-related, or a result of systemic inflammation. However, inflammation in biopsies early after transplantation has been connected to the development of dnDSA and the progression of fibrosis (17). The role of subclinical donor-related findings is not well studied, however, in one study mild pathological findings in time-zero biopsies did not affect early kidney graft function (31). In our study, the systemic pathway-specific inflammation scores correlated with inflammatory findings both early and 1 year after transplantation, and both systemic and graft-specific inflammation at 6 weeks were risk factors for inflammation in biopsies 1 year after transplantation.

In addition to the degree of inflammation, the number of HLA-DR mismatches, multiple transplantations, PRA > 20%, low eGFR at baseline, presence of DSA, and smoking at the time of transplantation were positively associated with long-term kidney graft loss, as was younger age. This has been described previously in patients with late-onset antibody-mediated rejection (32). DGF and DSA are associated with an inflammatory environment and with reduced kidney graft survival (33–35), but in our analyses, the inflammation scores also remained significantly associated with graft loss when adjusted for these factors. End-stage renal disease is a complex condition that is also characterized by increased levels of inflammatory biomarkers. Although studies have suggested that restoration of kidney function after transplantation improves chronic inflammation (36), it is difficult to determine whether the inflammatory state 10 weeks after transplantation is reflective of the pre-transplant systemic inflammation or a result of post-transplant mechanisms. We have previously described an association between increased levels of markers for inflammation in the early phase after kidney transplantation and PTDM (25). PTDM has been found to be associated with overall kidney graft loss, but not necessarily with death-censored kidney graft loss (37). In our analyses, where the models were adjusted for systemic inflammation grade in addition to other relevant risk factors, there was no association between early PTDM and either overall- or death-censored graft loss, suggesting that the previously described association between PTDM and overall graft loss may be explained by subclinical inflammation.

The main strength of the study is the large number of included patients (699 kidney transplant recipients) without any “loss to follow-up” during the study period. All patients received a standardized short- and long-term follow-up program and all biomarkers were measured at a defined time slot after transplantation. The results were robust and consistent both when the inflammation scores were analyzed as continuous and categorical variables. An important finding is the correlation between systemic inflammatory biomarkers and findings in graft biopsies. When both the systemic inflammation scores and local inflammatory findings in the graft were included in the Cox regression models, the systemic inflammation scores remained significantly associated with graft loss. The observational study design is a recognized limitation, and although we have adjusted for confounders in the analyses, residual confounding is most likely present. We developed composite inflammation scores to avoid covariance and to develop more robust results, but mass significance is a risk when performing multiple tests on multiple biomarkers. We only have BMI for the population and not a representative marker of central obesity, like for instance waist-hip ratio, which may be more related to an inflammatory environment. For this population, we only have structured comorbidity data on diabetes and smoking status at the time of transplantation, and not for preexisting cardiovascular disease, which is an important potential confounder. We did not have sufficient data on CRP for this population, however, CRP is a pentraxin, and we have included PTX3, which displays many similarities with CRP. Also, the effect of reduced renal clearance on the biomarkers is not well described and this could potentially have influenced our results, although eGFR was used as a variable in the analyses (14).

In conclusion, based on total inflammatory- and pathway-specific scores, we have described a significant association between a systemic inflammatory environment early after kidney transplantation and long-term kidney graft loss. Vascular inflammation and increased fibrogenesis activity stood out among the tested pathways and were also associated with inflammatory- and fibrosis findings in biopsies.

## Data availability statement

De-identified data may be shared upon reasonable request and after application to the Regional Committee for Medical and Health Research Ethics (REK Sørøst, Norway) in cooperation with the authors.

## Ethics statement

The studies involving humans were approved by Regional Ethics Committee in Norway. The studies were conducted in accordance with the local legislation and institutional requirements. The participants provided their written informed consent to participate in this study.

## Author contributions

TH: Conceptualization, Formal Analysis, Investigation, Methodology, Writing – original draft, Writing – review & editing. AÅ: Conceptualization, Investigation, Supervision, Writing – review & editing. TU: Investigation, Methodology, Writing – review & editing. AR: Methodology, Writing – review & editing. SP: Writing – review & editing. TM: Methodology, Writing – review & editing. PA: Writing – review & editing. FR: Writing – review & editing. AH: Conceptualization, Supervision, Writing – review & editing. KH: Conceptualization, Project administration, Supervision, Writing – review & editing. TJ: Conceptualization, Investigation, Methodology, Supervision, Writing – review & editing.

## Supplementary material

Supplementary Figure 1 Violin plots displaying the distribution of the standardized values of the individual inflammatory biomarkers. The blue dots represent the median, and the orange diamonds represent the mean value.
