# Supplementary figures and images for: Systemic inflammation early after kidney transplantation is associated with long-term graft loss: a cohort study
Source: Front Immunol. 2023 Oct 2;14:1253991. doi: 10.3389/fimmu.2023.1253991 (PMC10577420; doi:10.3389/fimmu.2023.1253991)

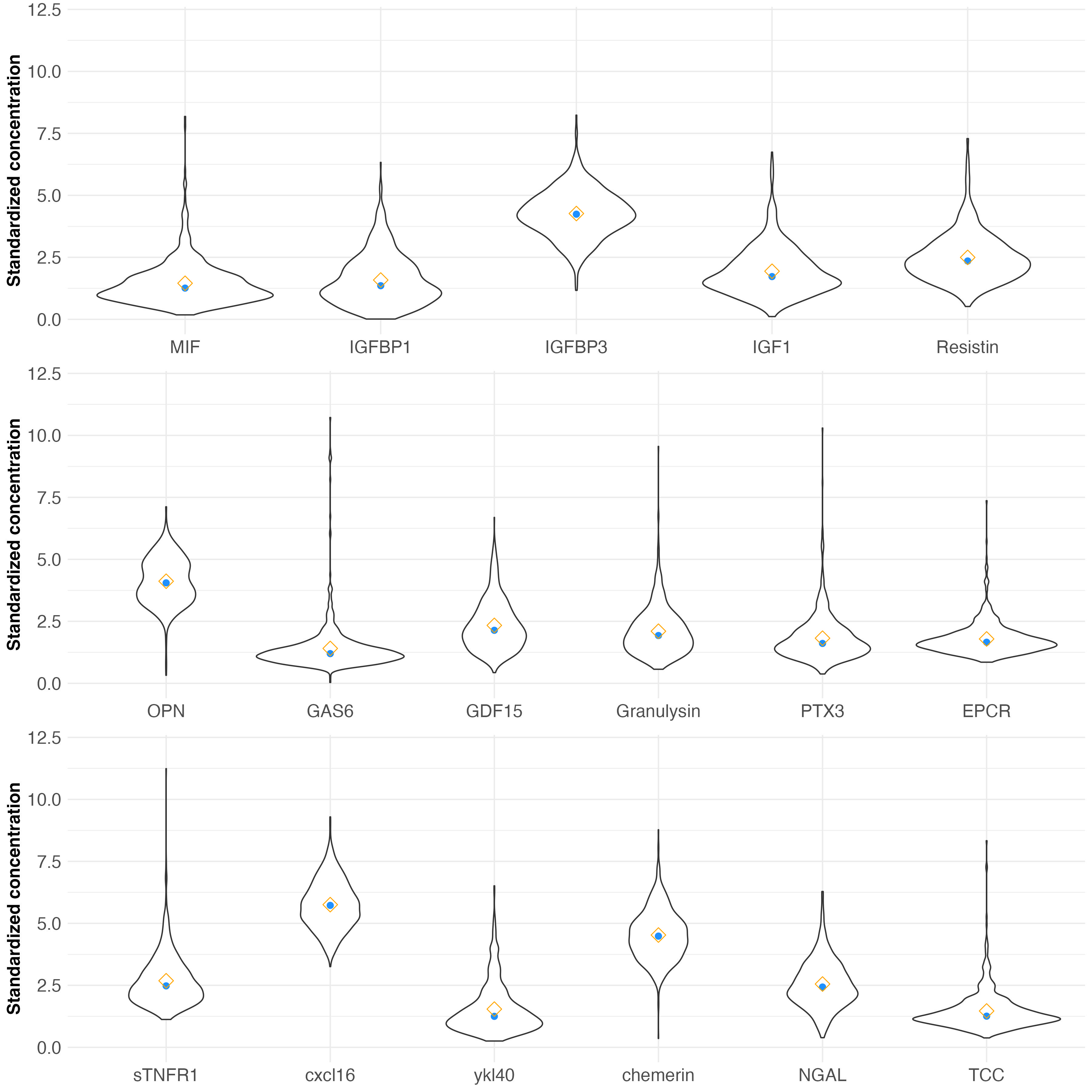

Supplement: Supplementary Figure 1 — Violin plots displaying the distribution of the standardized values of the individual inflammatory biomarkers. The blue dots represent the median, and the orange diamonds represent the mean value. [file Image_1.tiff]
